# Challenges and Opportunities for Mainstreaming Climate Change Adaptation into WaSH Development Planning in Ghana

**DOI:** 10.3390/ijerph14070749

**Published:** 2017-07-10

**Authors:** Salley Alhassan, Wade L. Hadwen

**Affiliations:** 1 International Water Centre, Australia, Oz Green Youth Leading the World (YLTW)-Africa Program, Ministry of Food and Agriculture, Berekum 233, Ghana; 2 Australian Rivers Institute and Griffith Climate Change Response Program, Griffith University, Nathan, Brisbane, QSD 4111, Australia; w.hadwen@griffith.edu.au

**Keywords:** climate change, adaptation, WaSH, policy, sustainability, development

## Abstract

Climate change threatens water, sanitation and hygiene (WaSH) facilities and services, as these are intimately linked to the water cycle and are vulnerable to changes in the quantity and quality of available water resources. Floods and droughts, which pollute and reduce water delivery respectively, have now become a perennial issue to deal with in the northern regions of Ghana. This study aimed to assess the degree to which climate change adaptation measures are mainstreamed into the water, sanitation and hygiene (WaSH) development planning process in Ghana. Stakeholders from government and non-government agencies were interviewed to gain perspectives on the threat of climate change, the inclusion of climate change in WaSH planning and the barriers preventing mainstreaming. Despite awareness of climate change, adaptation measures have not been considered, and the immediate WaSH needs remain the priority. Overall, stakeholders felt the adaptive capacity of the Municipality was low and that mainstreaming has not yet occurred. Despite the lack of progress, there are great opportunities for mainstreaming climate change adaptation into planning through increasing awareness and capacity, legislative and institutional changes and the development of participatory systems to provide early warning systems and disaster risk analyses that will inform future planning.

## 1. Introduction

Climate change has become one of the major issues threatening sustainable development in the world. Changes to weather patterns, including the reliability and predictability of seasonal rainfall and the impacts of extreme events, now place unprecedented pressures on water resources, especially in flood- and drought-prone regions of the globe. These growing water resource challenges make sustainable development objectives, especially those directly relating to water, sanitation and hygiene (WaSH) targets, even more difficult to achieve [1,2]. Importantly, the impacts of climate change are more severe in African countries than in other regions of the world [3], because they have weak climate change responses and adaptation strategies. There is widespread evidence that freshwater bodies are shrinking and losing their quality due to the current changing climate [4]. These changes make communities that depend on surface water vulnerable. Whilst significant levels of investment in WaSH in sub-Saharan Africa have achieved significant results during the Millennium Development Goal (MDG) period, the pressure of climate change threatens to undo previous interventions and hamper the continued progress that is required to satisfy the Sustainable Development Goal (SDG) agenda [2].

The impacts of climate-related disasters are well documented. For example, in recent years, the three northern regions of Ghana have experienced extreme climate change pressures such as droughts, floods and heavy storms. In 2007, these three regions experienced a serious flood which claimed 31 lives, displaced about 102,250 people, destroyed 39 dams, 45 schools, 58 bridges and culverts in the Upper East Region alone [5]. WaSH facilities such as public toilets and household latrines were also damaged. Furthermore, another series of floods in 2010 displaced 34,553 people and destroyed 5512 houses [6]. These pressures exacerbate the increasing poverty levels encountered in this region [7] and threaten the attainment of development goals.

The future projections for the climate in northern Ghana paints a grim picture; temperatures will rise by 2.1–2.4 °C and annual rainfall figures are expected to decrease by 1.1% by 2020 and 20.5% by 2080 [8]. These changes will directly and negatively impact the water cycle and affect the lives and livelihoods of people, especially the poor and the vulnerable in society.

Despite the recent climate events and the future projections for the climate, measures to mitigate and adapt to climate change in Ghana have been ad hoc (reactive) and largely in response to emergencies. As a result, the long-term consequences of climate change, and the associated anticipatory adaptation required to respond to these changes [9], are barely considered. Incorporation of adaptation measures into plans, programmes, projects and budgets can reduce the adverse impacts of climate change on the sustainability of development programmes and projects [10]. In this way, development interventions can become resilient through climate-proofing [11]. In a similar way, people’s adaptive capacities, particularly through their WaSH practices, can be improved and their vulnerability levels reduced through the integration of climate change adaptation into development plans [12]. Accordingly, there is a need to plan appropriately and pragmatically to embrace a development pathway that ensures resilience and incorporates climate change adaptation issues into all aspects of WaSH Sector development planning. Like many other developing countries, the challenge now lies with moving away from focusing solely on disaster and emergency WaSH relief, towards a forward-thinking strategic approach to WaSH development that is climate-resilient [13].

One way of integrating climate change adaptation into WaSH development policies is through the process of mainstreaming. Mainstreaming climate change adaptation is the process through which the risks of climate change are inherently built into the objectives of development projects [14]. Effective mainstreaming ensures that climate risks are incorporated into projects and policies in a way that will support long-term sustainable development. The concept of mainstreaming has been in existence since the 1990s and it has been used in the developing world to address issues such as gender inequality, disability, HIV/AIDS, poverty reduction and environmental degradation [15,16]. Mainstreaming captures potential across a range of policy areas through the implementation of projects which account for the cross-cutting issue of interest [17]. Conceptually, mainstreaming has many benefits, including increasing coherence across sectors, reducing duplication and cost of implementation, and minimizing the degree to which policies contradict each other [17]. The UN endorses the concept of mainstreaming for climate change adaptation and the approach is being used in many Non-Governmental Organisation (NGO)-supported projects around the world [12].

The widespread support for the concept of mainstreaming provides an opportunity for us to examine the degree to which climate change adaptation has been, or could be, mainstreamed into WaSH development planning in developing countries like Ghana. To date, integration of climate change into development programmes in Ghana has only been considered at the national level [18]. At the local level, it is not clear how or if district authorities are mainstreaming climate change adaptation measures into development plans. Indeed, as Nelson and Agbey [18], noted, climate change adaptation and its integration has only become a recent issue for policy-makers and planners in Ghana and its mainstreaming remains a challenge. Given this context, we explore the extent to which climate change adaptation measures are being mainstreamed into the WaSH development plans for sustainable development for the Bolgatanga Municipal Assembly, in northern Ghana. To do this, we interviewed government and NGO stakeholders and used a standardized questionnaire to gather information around WaSH development, climate change threats and risks and coordination of responses across all stakeholder groups. Based on our findings, we provide an approach which will aid in the conceptualization and mainstreaming of climate change adaptation into future WaSH policy, planning and implementation in the region.

## 2. Materials and Methods

### 2.1. Case Study Area—Bolgatanga Municipality in Perspective

Inadequate water supply and poor sanitation and hygiene practices remain a significant challenge throughout Ghana and many people suffer from water-related diseases such as malaria, diarrhoea and cholera as a result [19]. For the country to find and implement lasting solutions to these problems, it will be necessary to identify the main sources of water supply for communities and simultaneously examine aspects of their quality and quantity and threats to their safety in the context of consumptive use.

**Figure 1:**
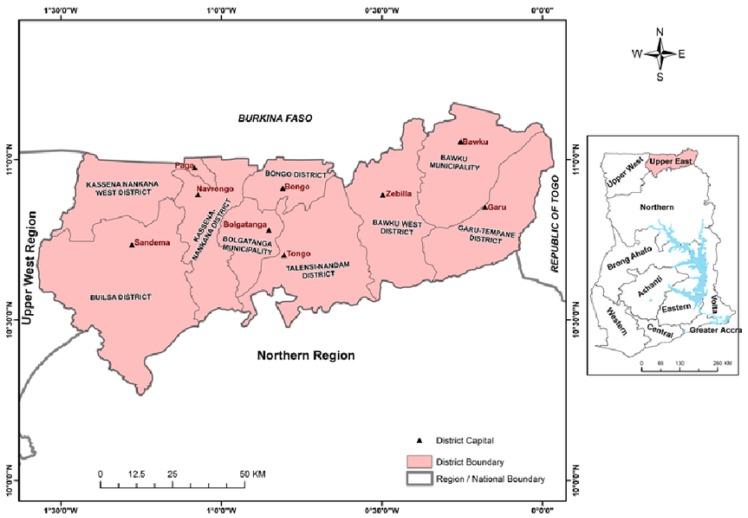
Map of Ghana Showing Upper East Region and its Districts including Bolgatanga Municipality. Source: [22].

Ghana is divided into ten regions which include the Greater Accra, Ashanti, Brong Ahafo, Central, Western, Volta, Upper East, and Upper West Regions. Governance takes place at the national, regional and district levels [20]. Bolgatanga is the regional capital as well as the administrative capital of the Upper East Region (see Figure 1). It is located within latitudes 10°30′ and 1°55′ north and longitudes 1°00′ west, with a total land area of 1620 km^2^ [21].

The Municipality experiences a unimodal and erratic rainy season starting from May/June to September/October with mean rainfall figures of between 800 mm and 1100 mm annually [23]. The rivers in the Municipality are the rivers White and Red Volta and Sissili, including their tributaries which serve as the main drainage for the area. From November to mid-February, the region experiences long dry spells with cold, dry and dusty Harmattan winds. During the Harmattan period, temperatures range from 14 °C at night and above 35 °C during the day. Daytime temperatures are usually high due to the low humidity [23].

### 2.2. Water Supply in the Bolgatanga Municipality

Surface water and groundwater sources are the main sources of water supply in Ghana [24]. The surface water supply includes rivers, streams and lakes while the groundwater supply includes hand-dug wells, boreholes and pipe borne water [24]. Within the Bolgatanga Municipality, households in the core of the three urban areas (Zuarungu, Sumbrungo and Bolgatanga) depend largely on piped water, supplied and managed by the Ghana Water Company Limited, (Bolgatanga, Ghana). In contrast, households in the peri-urban areas depend on boreholes and hand-dug wells, as these areas are not yet connected to the piped water system.

In rural areas, surface water (streams and rivers) and groundwater (borehole and hand-dug well) are the main sources of water available to communities. Traditionally, communities in rural areas depended largely on surface water from streams and rivers, but there is a growing trend for more reliance on borehole water in rural areas. This is due to the negative effects of pollution and droughts on the quality and quantity of surface water resources, and the efforts of the government of Ghana, led by Community Water and Sanitation Agency (CWSA) in partnership with NGOs, which have seen boreholes installed in most villages in the Municipality. Evidence suggests that this trend, away from the use of surface water sources, has reduced the incidence of water-related diseases in the Municipality [25]. However, despite the provision of boreholes, villages are still vulnerable to water shortages and water-related diseases due to the vagaries of the weather. Given the seasonal changes in water source quality and availability, most households depend on a mix of surface and groundwater supply systems depending on the geographical location.

### 2.3. Sanitation in the Bolgatanga Municipality

While an increasing number of Ghanaians (89%) have access to improved water sources, access to improved sanitation stands at 15% [26]. Within the Bolgatanga Municipality, urban areas with access to piped water tend to use water closet toilet facilities. In contrast, for the areas without piped water, particularly the peri-urban and rural areas, pit latrine and Kumasi Ventilated Improved Pit (KVIP) toilet systems are most common. In addition, some people within these communities resort to open defecation practices. This has remained a challenge in the Municipality, which is currently attracting attention from the Government of Ghana led by the Ministry of Local Government and Rural Development and international bodies such as the United Nations Children’s Fund (UNICEF) [27]. While this problem has been the main topic for discussion at various stakeholder meetings, open defecation is still widespread and is considered to be one of the major pollutants in water sources. Given this link, policies are often designed to tackle water supply and sanitation situations simultaneously. This is evident when the Government of Ghana implemented a policy in 1993 aimed to ensure the sustainability of water and sanitation facilities provided through a demand responsive approach [28].

### 2.4. Approach

To assess and understand stakeholder perspectives around climate change threats and mainstreaming into WaSH policy, planning and implementation, we adopted a qualitative research methodology. In assessing climate change adaptation strategies, qualitative research has numerous advantages. Principally, qualitative research focuses on the how (practices and processes) and not just outcomes or products [29]. Adopting the qualitative approach hence permitted a thorough analysis of participant’s experiences and views regarding mainstreaming climate change adaptation measures in Ghana. In addition, people’s views and experiences on climate pressures, capturing using a qualitative approach, are also useful in appreciating the various types of adaptation measures that may be required and accepted [30].

To collect our qualitative data, we purposefully identified 10 key stakeholders from institutions responsible for water management, disaster preparedness, and environmental management from the Bolgatanga Municipality (Table 1) for interviews using a semi-structured interview guide (interview questionnaire is presented in the Appendix A). This included stakeholders from the Municipality, from the National Government Departments and from two NGOs active in the region. The semi-structured interview protocol was reviewed and approved through the University of Queensland Ethics Committee (reference number CE16W226).

The stakeholder interviews were conducted between March and May 2016. Stakeholders agreed to participate freely in the study and were aware that they could withdraw their participation at any time. All of the interviews were conducted in person by Salley Alhassan and interview data was confidential and stored in a protected system known only to Salley Alhassan. In order to protect confidentiality and anonymity of interviewees, participant comments have been de-identified and coded.

The responses from the stakeholders provided in-depth information on how adaptation measures are mainstreamed at the policy-making level in the Bolgatanga Municipality. We used content analysis to systematically analyse the respondents’ responses to survey questions, principally through looking at who said what, to whom, why and to what degree and effect [31]. This enabled us to classify each interview script into parts that could then be treated as distinct units for thematic analysis. Qualitatively, each theme was evaluated to identify which of the themes most frequently occurred, in what perspective and how they are linked to each other. Some respondent’s answers were directly quoted to emphasize the argument being put forth around these emergent themes. Finally, we also obtained secondary information, to build on the stakeholder interviews, from the Municipal Medium-Term Development Plans, Municipal Annual Budgets, and Municipal Annual Reports.

## 3. Results

### 3.1. Stakeholder Views of Climate Change in Bolgatanga Municipality

The Bolgatanga Municipality, along with other areas in the northern part of Ghana, has been declared as a climate risk and disaster-prone area [32]. Stakeholders indicated changing rainfall patterns and increasing temperatures as the manifestations of climate change in the Municipality (Figure 1) and reported that the Municipality has been experiencing greater than expected variability in rainfall and higher temperatures, especially during extreme events. These changes in rainfall patterns, decreasing water quantity in streams and rivers and increasing temperatures were the most common climate change stressors in the Municipality. These stressors included heavy downpours causing floods or long dry spells resulting in droughts that often decrease the quantity of water in rivers, streams and lakes. The stakeholders indicated that there has also been a reduction in the predictability and reliability of seasonal weather patterns, to the point that flood or drought events can now occur at any time of the year. In general, the stakeholders expressed the view that climate change had led to the rapid depletion of natural resources due to farming activities, overgrazing, improper solid and liquid waste disposals, illegal mining and other anthropogenic activities around the Volta Basin. As mentioned by one of the stakeholders, “Due to the impact of climate change, people have turned to exploiting the ecosystem there by depleting natural resources”.

**Figure 2:**
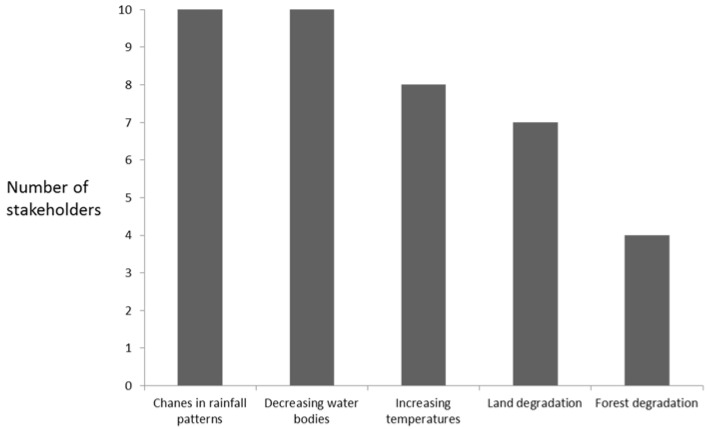
Perspectives on changing climatic conditions in the Bolgatanga Municipality, as indicated by interviewed stakeholders.

The stakeholders indicated that land and forest degradation represent some significant climate-related stressors which have also been experienced over the past years (Figure 2). This confirms the assertion that the vulnerability of an area to climate change exigencies is due to a number of inter-related factors such as reliance on rain-fed agriculture, less-developed water resources, land degradation and weak institutions [8].

The stakeholders agreed that the surface water sources including rivers and streams are shrinking in size (Table 2), which in turns affects the water level in the Basin and in the Vea Dam. It was also revealed that the rivers Red Volta and Sissili, both tributaries of the White Volta River, dry up for approximately two months a year due to drought [33].

It is important to note that climate change affects not only surface water but groundwater as well. An interview with the Municipal Planning Unit revealed that some boreholes have already dried up and many more are becoming dysfunctional; this is widely attributed to the impacts of climate change. It was added that in the past it could take only forty pumps to fill-up a 40 L bucket, but with the current drought conditions, 60 pumps are now required. This supports the view that the groundwater table in the Municipality was dropping [34]. Rural Aid responded that more than half of water resources in the Municipality were not able to contain water all year round and this situation is expected to worsen further with climate change.

Beyond the impacts on water sources for WaSH, stakeholders also expressed the view that climate change is also impacting on food production, as farming is the channel through which climate change affects food security [35]. This was evident when one stakeholder said in an interview that; “Once water bodies are drying up, this will have negative impact on irrigation practices and subsequently our effort towards achieving food security”.

### 3.2. The Sensitivity of Social Infrastructure to Climate Change

Social infrastructure, including community buildings, can also be vulnerable to the impacts of climate change. The stakeholders revealed that floods, heavy downpours and long dry spells are the ways in which climatic change can affect social and community infrastructure (Table 3). Stakeholders revealed that houses, school buildings, toilet facilities and community health buildings are infrastructures that are both sensitive and vulnerable to climate change. Perhaps this is because most houses in the rural areas are constructed with mud and roofed with thatch or iron sheets. Moreover, communities along the White Volta Basin often experience flooding with buildings and other types of social infrastructure being affected. Thus, the local houses are very vulnerable to heavy rainfall and often during rainy season, many of them collapse. During the dry season, it is common to see people, especially women, rebuilding collapsed houses or maintaining damaged ones.

### 3.3. Climate Change Adaptation Initiatives in the Bolgatanga Municipality

Climate change is certainly a well-known problem in Ghana, with almost all of the stakeholders having participated in climate change stakeholder capacity-building programmes in the last four years. Encouragingly, stakeholders indicated that climate change discourses have focused on awareness campaigns and adaptation strategies. In particular, the Ghana Water Company Limited (GWCL) and the Water Resources Commission (WRC) have participated in climate change discussions that have focused on the impacts of climate change on water resources and climate adaptation measures such as riverbank restoration. This shows that these institutions have some basic knowledge of climate change and its impacts and risks, as well as some relevant adaptation and mitigation measures. However, despite many efforts to address climate change, stakeholders commented that there are still major barriers and gaps to address to ensure sustainable outcomes—these will be addressed in the following sections.

### 3.4. Vulnerability Assessment

Climate change vulnerability assessments predict both the current and future effects of climate change, the vulnerabilities of communities and ecosystems to these effects, and the measures that support adaptation to all the impacts [36]. Climate change vulnerability assessment is crucial in designing adaptation measures, particularly in communities where livelihoods are predicated on natural resources such as water and ecosystem resources. The failure to undertake vulnerability assessments will affect capacities to adapt to climate change impacts. This is clearly the case in the Bolgatanga Municipality, as it is evident that vulnerability assessment and WaSH delivery in the Municipality remains a great challenge. While some of the stakeholders indicated that climate change vulnerability has not been assessed, others are uncertain about whether this activity has been carried out (Table 3). They advised that departments directly involved in WaSH activities should be contacted for further information on climate change vulnerability assessment. The stakeholders who agreed that climate change vulnerability assessment had been carried out, could not measure or articulate the outcome of the assessment (Table 4). This can be juxtaposed with Oates et al. [15], who stated that integrating climate change adaptation into mainstream development policy at the local level is less of a priority than the national-level initiatives. In a related way, the United Nations Environment Program-United Nations Development Program (UNEP-UNDP) Poverty–Environment Initiative [35] suggested that awareness and institutional capacity building are key entry points to begin climate change mainstreaming. It is important to note that support for institutional capacity, particularly for mainstreaming cross-cutting issues such as climate change, is yet to be achieved in the Bolgatanga Municipality.

### 3.5. Adaptation Programmes and Projects

The main adaptation programmes cited by stakeholders included the opening of drainages, dredging and rehabilitation of dams, clean-up campaigns and use of drought resistant crops. These measures are implemented by the Bolgatanga Municipal Assembly in response to the current threats, including the impacts of climate change. In addition, the Environmental Protection Agency (EPA) has implemented related programmes of work which seek to diversify livelihoods from traditional agriculture to other ventures, restore riparian buffer zones and tree planting activities. Some of these activities, like riverbank restoration and riverbank alternative livelihood support are also undertaken, separately, by the Water Resources Commission (WRC). Despite these efforts towards addressing and adapting to climate change, there are still gaps to be addressed mainly because the programmes and projects lack proper coordination at the assembly level. While some stakeholders could not mention any adaptation measures put in place by the Municipal Assembly as a whole, others felt that coordination of these programmes at the assembly level was either poor or non-existent.

Questions regarding the resourcing of adaptation programmes revealed that the main source of funding for adaptation activities for the Municipal Assembly were from the Internally Generated Funds (IGF), whereas funding for the decentralised departments and their activities were mostly from donor agencies. This leads to some lack of coordination in adaptation response. For example, there was no alternative livelihood programme implemented by the Municipal Assembly to adapt to climate change, whereas programmes in this space were promoted by institutions such as WRC and EPA and mainly sponsored under development assistance.

### 3.6. Mainstreaming Adaptation Policies into WaSH Development Planning

To effectively mainstream adaptation into development planning, institutions must fully understand climate change issues and thereby discover the right areas for intervention [37]. As such, strengthening the capacity of institutions is critical to achieving successful mainstreaming of climate change adaptation issues into policy planning [38]. Interestingly, there is evidence of this strengthening occurring in Bolgatanga, with stakeholders who attended more climate change capacity-building programmes indicating that they had begun to incorporate climate change adaptation measures into their planning processes. However, it is also important to note that some stakeholders are not fully aware of the adaptation measures being implemented—many of them said that the Municipal Assembly should be contacted regarding climate change adaptation issues. This might be attributed to a lack of institutional coordination and collaboration, particularly at the Municipal level. The risks associated with poorly coordinated approaches to adaptation are that maladaptive measures may be implemented, as the actions at one level might have negative consequences at other levels of the system.

Though institutional capacity is important for mainstreaming climate change adaptation into policy planning, it is not limited to only capacity building. It is also important to develop and strengthen the structures for institutions to handle climate change issues. The necessary institutional arrangements required for mainstreaming climate change have not yet been implemented by the Municipal Assembly. In addition, it is clear that climate change issues are added to existing roles and responsibilities, as neither the Assembly nor the decentralized departments had a desk officer or focal person in charge of climate change issues.

Municipal Development Plans represent an access point through which climate change adaptation can be mainstreamed. The Medium-Term Development Plan (MTDP) prepared by the Municipal and District Assemblies (MMDAs) in Ghana is used as a working document responding to the development challenges of the area for a period of time. The MMDAs are required to develop their action plans to harmonize with the National Medium Term Development Planning Framework (NMDTPF). This means that the Assembly’s development plans and budgets should be in sync with the seven thematic areas of the NMTDPF, which includes crosscutting issues such as climate change, gender and disability. For the Bolgatanga Municipal Assembly, the MTDP for 2014–2017 based on the Ghana Shared Growth Development Agenda (GSGDA) is yet to be finalized [39]. This therefore presents an opportunity for the Municipality to capture and highlight the emerging climate change and WaSH issues and to mainstream them into the plan. Taking these types of opportunities is really important, as failure to recognize these important issues will result in a lack of resources to support adaptation and mainstreaming efforts. For example, analysis of the Composite Budget of the Bolgatanga Municipal Assembly for the 2015 Fiscal Year indicated that there was no direct climate change adaptation strategy to be followed in response to prevailing or anticipated climate change impacts. Moreover, there were seven projects related to environment, water and sanitation from the 2015 budget. These projects included public education on environmental cleanliness, monthly clean up exercises, improvements to waste management, support for soil improvement activities, provision of boreholes to communities, installation of small town water systems for selected communities and the construction of water closets and KVIP toilets [39]. In the view of the Environmental Health and Sanitation Unit (EHSU), climate change issues were completely missing in the 2015 Budget. The EHSU stakeholder clarified further that, aside from the small number of environmental issues captured in the 2015 Annual Action Plan; there was no talk about climate change in the plan.

The stakeholder from Water Vision Technology agreed with the assertion that climate change was absent in the budget and hence tried to differentiate between environmental concerns and climate change issues. The institution emphasized that it is hard to dissociate climate change adaptation measures from measures pursued to improve the environment and added that the difference depends on the reasons behind the development of these measures. Hence, adaptation measures are intended to mitigate particular impacts of climate change and increase resilience against some forms of vulnerabilities. On the other hand, environmental measures are targeted at tackling particular environmental issues such as land degradation, which may not be specifically caused by climate change. It is important to add that climate change is likely to make environmental problems worse, which in turn suggests that mainstreaming is still required.

Although some of the stakeholders indicated that they incorporate climate change into their action plans, there had not been any vulnerability assessment or impact assessment of these projects. There could be many reasons for this inaction. As indicated by the Planning Unit, the tree planting and public education on water pollution initiatives were not fully implemented because funds were not flowing regularly and this has slowed progress substantially. It was also revealed that there was no assessment to determine the impacts of these initiatives in the Municipality.

### 3.7. Mainstreaming Climate Change: Challenges and Prospects in the Bolgatanga Municipality

A number of challenges regarding the mainstreaming of climate adaptation into WaSH development planning were identified during the stakeholder interviews. These comprised the non-existence of institutional structures and constitutional support, lack of coordination and collaboration, inadequate information on the districts vulnerability to climate change, inadequate early warning system strategies and the trivializing of climate change issues. These challenges arise because different institutions are responsible for different aspects of the environment without proper institutional coordination at the decision making and policy implementation levels. All of these issues highlight how Ghana’s climate change adaptation behaviour is typical of a developing country with poor adaptive capacity and low political influence and will. Nevertheless, there has been a series of inter-institutional dialogues and discussions, often organized by external institutions including UNDP and Care International [20]. These dialogues provide a common platform to discuss the impact of climate change in the northern part of Ghana and represent an opportunity for building understanding and capacity and this is an important first step towards mainstreaming.

#### 3.7.1. Limited Information on the Municipality’s Vulnerability to Climate Change Effects

Stakeholders identified that information, particularly scientific information, was virtually not available in the Municipality. Though climate change issues such as flooding and drought are a perennial issue in the Municipality, there has not been any vulnerability assessment in the area not has there been shared information on likely climate driven changes in intensity and frequency of these events. The NGOs interviewed indicated that they did not have any scientific information to enable discussion of the specific areas of the district that are vulnerable to climate change. The Municipal planning unit and the EPA also indicated that there has never been any assessment of the district’s vulnerability to climate change. The United Nations Framework Convention on Climate Change (UNFCCC) and the Intergovernmental Panel on Climate Change (IPCC) agree that effective planning is reliant on the understanding of what is been planned and considering climate change adaptation, a risk assessment of current and future impacts of climate change and vulnerability conditions is critical in enabling adaptation and building resilience [40,41]. Without these processes, climate change adaptation actions become reactionary and not at all mainstreamed.

#### 3.7.2. Non-Existence of Institutional Structures and Statutory Support at the Municipal Level

Establishing institutional structures is key in advancing the mainstreaming of climate change adaptation. In the National Medium Term Development Plan Framework (NMTDPF), cross-cutting issues such as gender and disability have been identified, together with climate change, to be mainstreamed into the District Medium-Term Development Plans (DMTDP). While gender and disability have been mainstreamed into the administrative structures of the Assembly, climate change is yet to receive such support. The EHSU and CWSA stakeholders suggested that the Municipal Assembly needs to have a climate change desk officer to promote and coordinate climate change issues. Furthermore, there has not been any statutory support for the mainstreaming of climate change and this is not only at the district levels but the national level as well [42]. In the Bolgatanga Municipal Action Plans, there have been no specific funds allocated to support climate change adaptation programmes. This is, in part, due to the fact that the Assembly, by law, is not obliged to devote any funds to climate change. As a result, mainstreaming climate change issues into development planning is left as a choice in the Municipal Development Planning Agenda. The EHSU and CWSA contended that without a law obliging the District Assemblies to include climate change issues and threats, this will remain an issue for discussion without concrete actions to address it. The GWCL indicated that statutory support should be created to allow the district assemblies to commit a percentage of the District Assembly Common Fund (DACF) to fund climate change adaptation, just like the two percent allocation currently permissible for disability projects. GWCL added that unless this is done climate change issues will always be underestimated and will remain under resourced at the district level.

#### 3.7.3. Inadequate Early Warning Systems Strategy

It is very important to share meteorological information, including climate change projections to communicate reliable information on weather conditions and threats. This is very relevant for monitoring specific climate change stressors such as floods and drought and enables the preparation of early warning measures necessary for households and farmers. An example of this comes from NADMO, who mentioned that every year, at the beginning of the rainy season, they sensitize communities in the Municipality about potential risks such as floods. This was confirmed by the Municipal Planning Unit in a similar claim that households are usually informed through announcements on radio stations particularly about the opening of the Bagre Dam Spillway in Burkina Faso, so that they can prepare against the potential risks.

In contrast, the Planning Unit added they are sometimes taken by surprise as they are not always able to determine the severity of the floods. This problem is also compounded by the fact that sometimes the Burkina Faso Authority does not communicate with them before opening the Dam’s Spillway. This indicates that there is a potential transboundary issue looming between Ghana and Burkina Faso. It is essential to add that recently, efforts have been made by both countries to improve communication concerning the opening of the Dam Spillway [20].

#### 3.7.4. Shifting the Responsibility for Climate Change Issues

From the interviews, it could be inferred that for some stakeholders, climate change adaptation issues were viewed as someone else’s responsibility. It was common to record comments such as “contact Municipal Assembly”, “refer to Municipal Assembly” for information regarding climate change in the Municipality. This issue could be as a result of poor coordination and collaboration between departments in the Municipality. As one stakeholder put it; “Collaboration is only seen at review meetings and that is when we seem to share information to each other”.

In contrast, the Water Resources Commission (WRC) emphasizes the need for collaboration with other ministries, departments and agencies as integrated water resources management affects various aspects of the society [43]. It also emphasized the need for effective collaboration especially with key stakeholder in their operational areas.

Notwithstanding the challenges recounted above, there are prospects for incorporating climate change into WaSH development planning in the Bolgatanga Municipal Assembly. From the view of the stakeholders, these prospects include capacity building, vulnerability assessments, environmental impact assessments and institutional reform to integrate and mainstream climate change adaptation.

#### 3.7.5. Capacity Building

The understanding of climate change adaptation by policy makers is essential in mainstreaming these issues into programmes. The stakeholders indicated that there should be training programmes for all the staff in the Municipal Assembly and other non-state actors such as NGOs. This is required to build their capacity not only on issues relating to climate change but also around how to effectively integrate climate change adaptation into WaSH development planning.

#### 3.7.6. Vulnerability and Disaster Risk Assessments

Despite the recognition of extreme events and the threat that climate change poses, it is clear that vulnerability or disaster risk assessments in the Municipality have not been carried out. Most stakeholders emphasised the need to carry out a thorough vulnerability or disaster risk assessment, ahead of developing strategies to mainstream climate change adaptation into planning. In this regard, such an undertaking would underpin the design of policies that will reduce the impacts of disasters, pollution and destruction of WaSH facilities.

#### 3.7.7. Institutionalising Climate Change Adaptation within Governance Structures

The lack of administrative structures to support climate change adaptation, as reported by the stakeholders, makes it difficult for climate issues to be considered in policy discourse. As mentioned previously, stakeholders suggested that District Assemblies need to have climate change desk officers who will advocate and coordinate climate change adaptation issues. The stakeholders argued that gender and disability issues have been successfully mainstreamed because there are administrative and governance structures supporting them. In this sense there is a case for a closer examination of the mainstreaming of gender and disability issues, in order to learn about the approaches that have been taken and to adopt one that will most likely be successful for achieving the climate change adaptation mainstreaming.

## 4. Discussion

### 4.1. Challenges and Prospects of Mainstreaming Climate Change into WaSH in Bolgatanga Municipality

Water supply, sanitation and hygiene delivery continue to remain a challenge in the Bolgatanga Municipality. At present, service provision can only be described as basic [2]. Climate change is known to place further pressure on water resources and despite the urgency and scale of these challenges, it is clear that the Municipality has weak responsive mechanisms when it comes to responding to climate change threats. Furthermore, all of the adaptation measures that have been implemented have been reactive and in response to immediate threats from extreme events and have not been implemented on the basis of the Municipality’s prevailing and increasing vulnerability to floods and droughts. Finally, though there have been several climate change capacity training programs, there is no evidence of climate change mainstreaming in the Municipal Action Plan.

There are many barriers which prohibit the mainstreaming of climate change adaptation issues into WaSH development plans in the Bolgatanga Municipality. First, there is inadequate information about the Municipality’s vulnerability to climate change and its hazards. Second, there are no institutional structures in place to mainstream climate change adaptation into the Municipal Assembly plans and there is no coordination and collaboration between stakeholders in the Municipality, making advocacy and coordination of climate change issues difficult. It was also observed that WaSH professionals in both governmental and non-governmental organisations were interested in building WaSH facilities and not really concerned about the impact of climate change on these facilities, probably due to the inadequate knowledge in climate change issues. This could also be exacerbated by politics, where the government is mostly interested in commissioning boreholes and toilets constructed for political gains. Also, livelihood interventions were mainly carried out by few of the decentralised institutions and these interventions were mostly sponsored by external donors. There is a growing concern that the sustainability of these projects, or lack thereof, will become a problem after the sponsors have left. On the basis of suggestions from the key stakeholders in the WaSH sector interviewed in this study, the following strategies are recommended to support the mainstreaming of climate change adaptation into WaSH development planning for the Bolgatanga Municipality.

#### 4.1.1. Capacity Building

As indicated by Huq and Ayers [35], the mainstreaming process should begin with building awareness and capacities of all stakeholders involved in the management of resources in the Municipality. Therefore, it is recommended that the local government should organise training sessions on climate change adaptation for Municipal Assembly staff, decentralised departments, NGOs and community elders. Through these training workshops, stakeholders will be trained on how to effectively mainstream climate change issues at the local levels which are then harmonised and coordinated at the regional level and up into the national plans i.e., the NMTDPF. This will ensure a move away from the traditional top-down approach to the mainstreaming of cross-cutting issues including climate change.

#### 4.1.2. Institutionalising and Coordination of Climate Change

There are currently virtually no structures put in place to mainstream climate change and mainstreaming cannot take place in an institutional vacuum. Therefore, it is recommended that administrative and governance structures through the Ministry of Local Government and Rural Development (MLGRD) should create desk offices for climate change particularly at the district levels that will be in-charge of integrating and coordinating climate change adaptation issues across all sectors of the Assembly. The desk officers will ensure effective collaboration between the various stakeholders including governmental, non-governmental and decentralised departments.

#### 4.1.3. Legislative Support for Mainstreaming Climate Change

At the national level, climate change has been incorporated into the national development policy. The approach to this incorporation has been top-down in nature and at the local level, there is no legislative support for mainstreaming climate change. Therefore, it is recommended that the government, through legislation, should oblige MMDAs to allocate a certain percentage of their revenue or the DACF to fund climate adaptation initiatives.

#### 4.1.4. Establishing Early Warning Systems and Disaster Risk Readiness

The lack of early warning systems and disaster risk readiness has further weakened the adaptive capacity of the Municipality. Hence, to ensure effective mainstreaming, decisions must be based on climate change information that is reliable and represents a continuous assessment of climate threats, exposures and sensitivities. It is also vitally important that this information is broadly and clearly communicating to support and inform local level adaptation. The Municipality should invest in the development of early warning systems and awareness programmes, to ensure that stakeholders have easy access to relevant information around system specific climate change threats or disaster risks.

#### 4.1.5. Integrating Participatory Approach to Development Planning

To reduce local vulnerability, participatory approaches are required, as this enables the identification and selection of appropriate adaptation measures [44]. This approach contextualises and complements the development of policies from a bottom-up perspective. To address this, MMDAs should adopt a participatory approach which will ensure that indigenous knowledge regarding climate change impacts are captured and integrated into WaSH development planning. Ultimately, the sustainability of WaSH services and infrastructure in Bolgatanga Municipality will rely on the mainstreaming of climate change adaptation and greater shared understanding of the challenges that lie ahead.

### 4.2. Moving towards Mainstreaming

**Figure 3:**
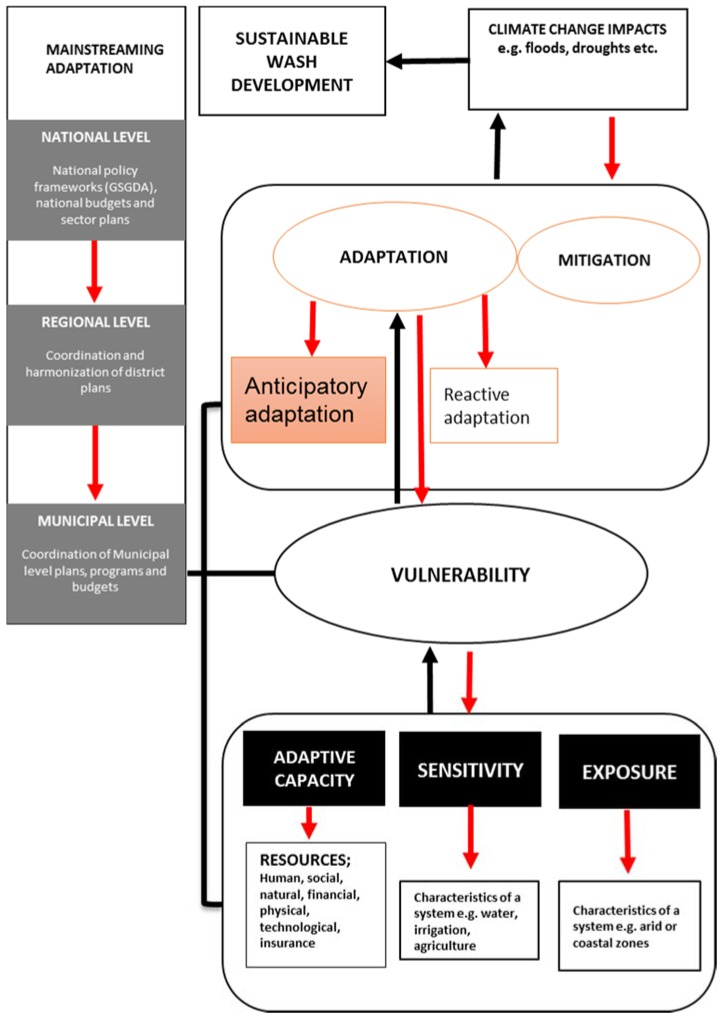
A conceptual model for mainstreaming climate change adaptation measures into development planning at all levels of government. The red arrows show the normal processes of adaptation, while the black arrows show feedback loops. Source: Authors construct, (2016). GSGDA is the Ghana Shared Growth Development Agenda.

WaSH development planning is a decision-making process involving a wide range of activities, at different institutional scales and across different developmental stages (Figure 3). At the national level, planning comprises policy formulation, development of national policies such as the Ghana Shared Growth Development Agenda (GSGDA) prepared by the National Development Planning Commission (NDPC), preparation of the national budget by the Ministry of Finance and Economic Planning (MoFEP) and preparation of other sectors plans and budgets. Similarly, work plans prepared by the districts are coordinated at the regional level in a manner that will ensure they are well-matched with National policy guidelines. Work plans at the district level are also developed to fit into the NDPC guidelines. With procedures provided by the Secretariat of the District Assembly Common Fund, the district composite budgets are developed [39].

Figure 3 presents a conceptual model for mainstreaming climate change adaptation into development planning. The model presents two pathways; the normal processes of adaptation and the adaptation induced by planning. In the normal process (shown by the red arrows), climate change impacts are influenced by both adaptation and mitigation [45], although the focus of this model is on the adaptation component. It is important to note that adaptation may be reactive or anticipatory [46] and that decisions and outcomes will be influenced by vulnerability, which is a function of the adaptive capacity, sensitivity and exposure of the system.

The process of mainstreaming includes the integration of climate change adaptation issues into WaSH sector policies, plans and budgets at these national and municipal levels [16]. Once this has been achieved, the coordination and harmonization processes will need to take place at the regional level. To this end, the black arrows and lines in the conceptual model shows the relationship between policy at multiple levels and adaptation actions and how mainstreaming these policies will support better adaptation outcomes (more anticipatory actions shown in bigger box).

## 5. Conclusions

The Bolgatanga Municipality is highly vulnerable to the impacts of climate change, especially those which threaten the security of water resources. These impacts will significantly affect the capacity of the government to deliver basic WaSH services. Furthermore, current efforts to address climate stressors are too short-sighted and reactive to achieve lasting progress towards the sustainable development goals for water (SDG 6).

Mainstreaming climate change adaptation into WaSH development planning in the Bolgatanga Municipality offers a path forward, both to strategically respond to the climate change threat and to deliver the sustainable WaSH services required by the communities in this region. Mainstreaming is particularly powerful in that it can lever political will and resources across multiple sectors [17], which will not only have multiple benefits for the people of Ghana, but will also reduce the likelihood of management decisions being maladaptive and causing unintended consequences for the people in urban and rural communities.

Importantly, there remains an opportunity to influence the yet-to-be finalised Medium-Term Development Plan (MTDP) for the Municipal Assembly to ensure that climate change adaptation and WaSH issues are mainstreamed to achieve a wide range of development objectives. Learning from recent experiences around mainstreaming gender and disability into planning processes, an optimistic view should be taken towards mainstreaming climate change into Ghana’s planning policies in the near future. Successful mainstreaming of climate change will support improved outcomes across multiple sectors as well as achievement of the sustainable development goals.

## Figures and Tables

**Table 1:** Institutions selected for interviews, on the basis of their responsibilities for water management, disaster preparedness, and environmental management in the Bolgatanga Municipality. WaSH: water, sanitation and hygiene.

**Municipal Assembly Staff**	Municipal Budget Unit (MBU) Municipal Planning Unit (MPU)
**Heads of Departments Responsible for Water Resources Management and Sanitation Delivery**	Ghana Water Company Limited (GWCL) Community Water and Sanitation Agency (CWSA) Water Resources Commission (WRC) Environmental Health and Sanitation Unit (EHSU) Environmental Protection Agency (EPA) National Disaster Management Organization (NADMO)
**Non-Government Organisations in WaSH Delivery**	Water Aid Water Vision Technology

**Table 2:** Stakeholder perspectives of the impact of climate change on water resources.

Stakeholders	Responses	Themes
**Municipal Planning Unit**	Water sources drying up more than before and addition to climate change, dry season farming is also a contributory factor. Thirteen boreholes dried up.	water drying up
**Municipal Budget Unit**	Water sources drying up rapidly.	water drying up
**EPA**	Most of the rivers and dams that were hitherto not drying now dry up quickly. Farming activities along riverbanks and building in waterways.	water drying up
**NADMO**	Some water bodies dry out during the dry season. Unplanned building structures and cultivation of crops.	water drying up
**WRC**	Excessive variation between water availability during the dry season and rainy season.	water drying up
**EHSU**	The water sources are now drying up quickly.	water drying up
**CWSA**	The high temperatures being experienced are drying up the water bodies.	water drying up
**GWCL**	Water bodies are drying up. Groundwater is particularly more vulnerable.	water drying up
**Water Vision Technology**	Both surface and underground water bodies are depleting.	depleting water bodies
**Water Aid**	Reduced water levels especially during the dry season when water in the rivers and dams are used for dry season vegetable production.	water drying up

**Table 3:** Stakeholder perspectives of the impact of climate change on social infrastructure.

Stakeholders	Direct Quotes from Respondents	Themes
**Municipal Planning Unit**	Most houses in the rural areas are built with mud and roofed with local materials. These buildings are easily destroyed when there are floods or heavy downpour.	floods or heavy downpour issues
**Municipal Budget Unit**	There are reported cases of schools´ roofing sheets and sign boards being removed by heavy windstorms.	heavy windstorms issues
**EPA**	Most of the buildings in the area have their roof been ripped off because of increase in wind speed and intensity. Flash floods also destroy homes.	High wind speed, intensity and flash floods
**NADMO**	Flooding has become an annual ritual affecting the Municipality. Homes and schools in flood-prone areas are affected anytime there are heavy downpours.	flood issues
**WRC**	High demand for water, especially during dry season, affects the sustainability of social infrastructure such as water facilities.	dry season issues
**EHSU**	Heavy windstorms destroy the roofs of some toilets in the Municipality.	heavy windstorm issues
**CWSA**	Every year many households suffer damage during floods.	flood issues
**GWCL**	Most local houses are worn out by the excessive downpours and floods.	flood issues
**Water Vision Technology**	Social infrastructure such as schools, community health post and homes are in danger due to the changes in climate.	climate change issues
**Water Aid**	Rural infrastructure especially houses made of mud and roofed with thatch are the most affected when there are heavy rains.	heavy downpour issues

**Table 4:** Awareness of stakeholders regarding vulnerability assessments of climate change and WaSH in the Municipality.

Stakeholders	Responses		
	Yes	No	Direct Quotes from Respondents
**Municipal Planning Unit**	√		Cannot measure the outcomes. Maybe you need to contact the CWSA.
**Municipal Budget Unit**		X	Assessments are not related exactly to WaSH.
**EPA**	-	-	Contact the CWSA.
**NADMO**	√		The public now knows the need to protect the environment.
**WRC**	√		Development of WaSH strategic plan marketing for the Municipality by an NGO. The WRC has developed an Integrated Water Resources Management (IWRM) plan for the White Volta Basin
**EHSU**	-	-	I am not able to vividly mention whether there has been any vulnerability assessment in the Municipality
**CWSA**	-	-	Contact NADMO
**GWCL**		X	
**Water Vision Technology**		X	
**Rural Aid**		X	

## Appendix A

**Interview Guide for Municipal Assembly Staff, Heads of Departments/Agencies and NGOs**

**Mainstreaming Climate Change Adaptation into the Wash Sector Plans for Sustainable Development in the Bolgatanga Municipality**

Date of Interview: ................................. Contact number ………………………..

### Appendix A.1. Section A: Current State of Water Supply, Sanitation and Hygiene in Bolgatanga Municipality

1. What are the main sources of water supply in the Municipality?…………………………………………………………………………….
2. Is water available to people’s homes? How far do some people have to go get water?.....................................................................................................................................
3. Do any of the water sources in the municipality dry out? If yes, when and for how long?.........................................................................................................................................
  - Are the available water sources of sufficient quality for drinking?....................................................................................................................
  - Are some of the water sources more polluted or contaminated than others?.........................................................................................................................
4. What are the issues (threats/pressures) relating to water quantity and quality for people? ………………………………………………………………………………………
5. What types of sanitation are used by people in the Municipality?.................................
6. Are there areas or group of people that do not have adequate access to improved sanitation?................................................................................................................................
7. What is the normal practice of solid waste disposal for the affected population?
  - Refuse pit?
  - Public dump?
  - Bins?
  - Others?………………………………………………………………………………

### Appendix A.2. Section B: Vulnerability of the Municipality to Climate Change

1. What are some of the changes the Municipality experienced since the past ten years? *(Use the following as prompts)*
  - Rainfall patterns? (Droughts, floods and disease outbreak)
  - Temperature?
  - Water bodies? (Rivers, streams, dams etc.)
  - Soil Degradation?
  - Land Degradation?
  - Forest Degradation?
  - Others?........................................................................................................................
2. Describe the impact of climate change on the following in your municipality;
  - Water resources.........................................................................................................
  - Irrigation Agriculture……………………………………………………………..
  - Human health and security including sanitation………………………………
  - Social Infrastructure.................................................................................................
  - The environment........................................................................................................

### Appendix A.3. Section C: Adaptation Initiatives

1. Within the last four (4) years, have you attended any programme on climate change?....................................................................................................................................
  - If yes, how many programmes?..............................................................................
  - Briefly state what you have learnt from the programme(s)?.............................
2. What is your understanding regarding climate change?..............................................
3. Has there been any assessment of the vulnerability of climate change and WaSH in the Municipality?...................................................................................................................
  - If yes, briefly state the outcomes/findings (*ask for a copy if available*)…………
  - If no, explain why?...................................................................................................
4. Does the municipality have a disaster risk reduction (e.g. flood? Draught? Or disease outbreak?) strategy?................................................................................................
  - If yes, briefly describe the strategy (*ask for a copy if available*)…………………
5. What adaptation programmes and projects have been put in place by the Municipal Assembly over the past years (*may be 5 years or more*)?.................................
6. Are there other programmes and projects that have been carried out by other organisation other than the Municipal Assembly?............................................................
  **Table:**
  Organisation	Focus Area	Adaptation Initiatives	Duration of Programme/Project
  1.			
  2.			
7. Were the initiatives coordinated by the assembly? If yes, how was it coordinated?………………………………………………………………………………..
8. Are there any funds allocated for tackling climate change threats as and when they occur? Could you please mention examples?...............................................................................................................................

### Appendix A.4. Section D. Mainstreaming of Adaptation Policies

1. Do you incorporate climate change issues in Water resources management in the Municipal Medium Term Development Plan? What climate change issues have been captured in the Municipal Development Plans?......................................................................................................................................
  - If yes, could you please give examples? (Ask for the copy of MMTDP if available)……………………………………………………………………………
  - If no, give reasons why?............................................................................................................................
2. Describe the funding arrangements for these initiatives………………………….…….
3. Has the initiative been fully implemented? What percentage? Could you explain why?........................................................................................................................................
4. Has the initiative had any effect on the vulnerability level of the Municipality?..........................................................................................................................
5. Could you please mention the challenges and prospects for mainstreaming climate change into development planning in the municipality?................................................
  - So far what has worked well?................................................................................
  - Could you explain why things worked well…………………………………..
  - So far what has not well?........................................................................................
  - Could you explain why things did not work well?.............................................
  - What measures needs to be put in place?.............................................................
